# Thermal and Flame Retardant Properties of Phosphate-Functionalized Silica/Epoxy Nanocomposites

**DOI:** 10.3390/ma13235418

**Published:** 2020-11-28

**Authors:** Il Jin Kim, Jae Wang Ko, Min Seop Song, Ji Won Cheon, Dong Jin Lee, Jun Woo Park, Seunggun Yu, Jin Hong Lee

**Affiliations:** 1 New Functional Components Research Team, Korea Institute of Footwear and Leather Technology (KIFLT), Busan 47154, Korea; ijkim@kiflt.re.kr (I.J.K.); jwko@kiflt.re.kr (J.W.K.); sonochemical@gmail.com (M.S.S.); jwcheon@kiflt.re.kr (J.W.C.); dongjlee@kiflt.re.kr (D.J.L.); 2 School of Chemical Engineering, Pusan National University, Busan 46421, Korea; 3 Next Generation Battery Research Center, Korea Electrotechnology Research Institute (KERI), Changwon 51543, Korea; parkjw@keri.re.kr; 4 Insulation Materials Research Center, Korea Electrotechnology Research Institute (KERI), Changwon 51543, Korea

**Keywords:** epoxy nanocomposite, flame retardant, click chemistry, SiO_2_ nanoparticles, phosphorus

## Abstract

We report a flame retardant epoxy nanocomposite reinforced with 9,10-dihydro-9-oxa-10-phosphaphenantrene-10-oxide (DOPO)-tethered SiO_2_ (DOPO-*t*-SiO_2_) hybrid nanoparticles (NPs). The DOPO-*t*-SiO_2_ NPs were successfully synthesized through surface treatment of SiO_2_ NPs with (3-glycidyloxypropyl)trimethoxysilane (GPTMS), followed by a click reaction between GPTMS on SiO_2_ and DOPO. The epoxy nanocomposites with DOPO-*t*-SiO_2_ NPs as multifunctional additive exhibited not only high flexural strength and fracture toughness but also excellent flame retardant properties and thermal stability, compared to those of pristine epoxy and epoxy nanocomposites with a single additive of SiO_2_ or DOPO, respectively. Our approach allows a facile, yet effective strategy to synthesize a functional hybrid additive for developing flame retardant nanocomposites.

## 1. Introduction

Over the past decade, thermoset epoxy has been widely used in infrastructure and industry, including automotive, aircraft, aerospace and ocean, due to their advantageous mechanical properties, electrical insulation performances, thermal stability and chemical resistance [1,2,3]. Moreover, unlike other kinds of polymers, epoxy has the advantages of less shrinkage and the generation of a volatile substance during reaction, allowing for excellent processability [4,5,6]. Despite versatile properties of the epoxy, the intrinsically poor flame retardancy and stiffness have disturbed to widen the area in its practical application [7,8]. To improve the flame retardant properties of epoxy materials, bromination or chlorination approaches, which substitutes epoxy precursor with halogen atoms, have been used but causes fatal problem on generation of poisonous gas [9,10]. Meanwhile, phosphate compounds have received great attention as a promising flame retardant because the existence of phosphorus moiety significantly improve flame retardancy without release of any harmful gas [11,12,13,14]. However, the use of phosphorus flame retardant has still been suffered from accompanying deterioration of mechanical properties [15,16]. After advent of clay-type materials as flame retardant filler, 2-dimensional (2D) nanomaterials have received great attention to improve flame retardancy of epoxy resin by acting as physical barrier that can block the transfer of flammable gas and oxygen during combustion [17]. Representatively, layered double hydroxides (LDHs) composed of positively charged nanosheets and negatively charged interlayer can provide excellent thermal stability and flame retardant performances based on the structural characteristics [18,19]. Emerging 2D nanomaterials like reduced graphene oxide (rGO) and molybdenum disulfide (MoS_2_) are also beneficial for not only achieving flame retardancy but also simultaneously giving other functionalities such as mechanical, electrical and thermal properties [20,21,22]. In spite of the potential, the use of these kinds of 2D nanomaterials as flame retardant is limited due to the strong stacking of adjacent layers by the strong bonding by ionic or Van der Waals interactions, respectively. Recently, metal organic frameworks (MOFs) have been focused as new candidate of flame retardant for epoxy resin, in which the organic site can be compatible for polymeric matrix, while the metallic site can provide unique catalytic effect for efficient flame retardancy [23,24]. However, it is still required to improve the flame retardancy to be used as commercial flame retardant agent.

To improve the stiffness of epoxy with intrinsically high cross-linking density, incorporating of inorganic NPs -SiO_2_ for the almost cases-has been considered, giving rise to increased impact resistance [25,26,27,28]. For designing these types of composite materials, archiving homogeneous dispersion of the NPs within polymeric matrix is one of the most important factors because the NPs are likely to be aggregated by their high surface energy compared to that of polymer, which cause the inhomogeneity of mechanical properties of composites [29,30,31,32]. The SiO_2_ NPs are also useful to give the flame retardancy in polymer [33,34]. However, the use of the SiO_2_ NPs has a limitation on the improvement of flammability of the composite compared to other kinds of flame retardants because the mechanism is attributed to physical processes that improve the thermal durability by SiO_2_ NPs with high surface area and low thermal conductivity rather than chemical reaction [35].

One can expect that the use of hybrid additives of organic and inorganic materials would be beneficial to complement thermal and mechanical properties. Several researchers have designed the polymeric matrix modified with DOPO that is representative phosphorus flame retardant [36,37]. The DOPO-modified thermoset polymers, such as epoxy and polyimide, mixed with SiO_2_ NPs exhibited improved flame retardancy by the synergetic effect [38]. The DOPO capable of easily chemical modification was also advantageous to design organic-inorganic hybrid flame retardant as single filler for polymer nanocomposite. For instance, the DOPO could be easily grafted on the GO or MoS_2_ through solvothermal synthesis and the frame retardancy of their polymer nanocomposites was obviously improved [39,40]. Also, Q. Dong et al. reported that the hybrid, yet single filler of SiO_2_ directly tethered with DOPO improved flame retardancy as well as thermal oxidative stability of thermoplastic polypropylene (PP) [36]. However, although use of the DOPO improve the frame retardancy of the polymers, their mechanical properties are still suffered from the polymers plasticized by incorporation of DOPO [15]. Therefore, it is required to develop the new-type of filler based on DOPO with flame retardancy to complement degraded mechanical properties of the resulting polymer composites.

In this study, we fabricated flame-retardant epoxy nanocomposite with DOPO and SiO_2_ NPs as multifunctional additives. We first designed the hybrid additive of DOPO and SiO_2_ NPs by click chemistry strategy, providing high efficiency and yield in synthesis without major side reaction [41]. The functionalization of SiO_2_ NPs by commercially available 3-glycidyloxypropyl)trimethoxysilane (GPTMS) provided reactive site from epoxide ring, allowing for hybridization between SiO_2_ and DOPO via the click reaction to simultaneously improve mechanical properties and thermal stability of the epoxy nanocomposites with the DOPO-*t*-SiO_2_ NPs as single filler. The nanocomposites also exhibited the excellent flame retardancy without a loss of mechanical properties, which is analyzed through cone calorimeter and limiting oxygen index (LOI) test.

## 2. Experimental

### 2.1. Materials

Bisphenol A diglycidyl ether (DGEBA) with an epoxy equivalent weight (EEW) of 180~190 as epoxy resin was purchased from Kukdo Chemical in Seoul, Korea. Isophorone diamine (IPDA) as curing agent was purchased from Sigma Aldrich in St. Louis, MS, USA. DOPO as phosphorus flame retardant was purchased from Tokyo Chemical Industry in Tokyo, Japan. SiO_2_ NPs with a diameter of 5~20 nm as inorganic additive was purchased from Sigma Aldrich in St. Louis, MS, USA. GPTMS as coupling agent was purchased from Sigma Aldrich in St. Louis, MS, USA.

### 2.2. Preparation of GPTMS-Treated SiO_2_ (GPTMS-t-SiO_2_) Nanoparticles

As-received SiO_2_ NPs were used after drying at 120 °C for 24 h to eliminate moisture. SiO_2_ NPs of 5 g was dispersed in the mixed solvent of distilled water (DIW) and ethanol (EtOH) with 1:1 weight ratio by sonication for 30 min using horn-type sonicator (VCX 500, Sonics & Materials, Newtown, CT, USA), followed by vigorously mixing for 30 min at 25 °C. Sulphuric acid was added dropwise to the SiO_2_ NPs dispersion and the GPTMS of 0.5 g was added into the solution, followed by stirring for 24 h. After reaction, the GPTMS-*t*-SiO_2_ NPs was filtered using aspirator with vacuum pump, followed by repeatedly cleaning with EtOH 3 times and dried in oven at 70 °C for 24 h.

### 2.3. Preparation of DOPO-t-SiO_2_ Nanoparticles

DOPO powder of 3.28 g was put in the three neck reaction flask of 500 mL and heated in oil bath at 150 °C under N_2_ atmosphere for 1 h to melt DOPO. The GPTMS-*t*-SiO_2_ NPs of 0.6 g were added 5 times with the interval of 30 min and mixed for 3 h. After reaction, the DOPO-*t*-SiO_2_ NPs was filtered using aspirator with vacuum pump, followed by repeatedly cleaning with EtOH 5 times to remove residual DOPO and dried in oven at 80 °C for 24 h.

### 2.4. Preparation of DOPO-t-SiO_2_/epoxy Nanocomposites

A given amount of DOPO-*t*-SiO_2_ and DGEBA as epoxy resin of 40 g was mixed for 10 min at 25 °C, using a planetary centrifugal mixer (ARE 310, Thinky corporation, Tokyo, Japan) capable of shear mixing through rotation and revolution, in which the amount of the additives is expressed in parts per hundred of resin (phr). IPDA as curing agent of 9.3 g was added to the mixture and mixed for 20 min at 25 °C using the mixer, followed by defoaming using vacuum pump. As-mixed DOPO-*t*-SiO_2_/epoxy mixture was poured into Teflon mold with given size and shape, followed by curing at 120 °C for 2 h.

### 2.5. Characterization

Qualitative analysis of as-synthesized GPTMS-*t*-SiO_2_ and DOPO-*t*-SiO_2_ was performed using Fourier transform infrared spectrometer (FT-IR, FT/IR-6200, JASCO, Easton, MD, USA) in the wavelength range from 4000 to 650 cm^−1^. Flexural strength and fracture toughness of the composite samples were evaluated using a universal testing machine (UTM, DTU-90 OMHA, Dae Kyung Tech, Incheon, Korea) with a rate of 2 mm min^−1^ according to ASTM E399 and ASTM 399, respectively. Morphology analysis was performed using scanning electron microscope (SEM, JSM-6701F, JEOL, Tokyo, Japan) equipped with energy dispersive spectroscopy (EDS, X-MAXM, Oxford Instruments, Abingdon, Oxfordshire, UK). A cone calorimeter test was performed using a cone calorimeter testing machine (CC-105, Festec, Seoul, Korea) according to ISO 5660-1. After testing, the burning behavior of the samples was observed using a mobile phone camera (iPhone 10, Apple, Santa Clara, CA, USA). Thermal stability was investigated using thermogravimetric analysis (TGA, Q500, TA Instruments, New Castle, DE, USA) under N_2_ atmosphere. LOI testing was performed using an oxygen index meter (Oxygen Index, Fire Testing Technology, East Grinstead, West Sussex, UK) at 24.3 °C with a humidity of 20.1% according to ISO 4589.

## 3. Results and Discussion

### 3.1. Synthesis of DOPO-t-SiO_2_ NPs

**Figure 1:**
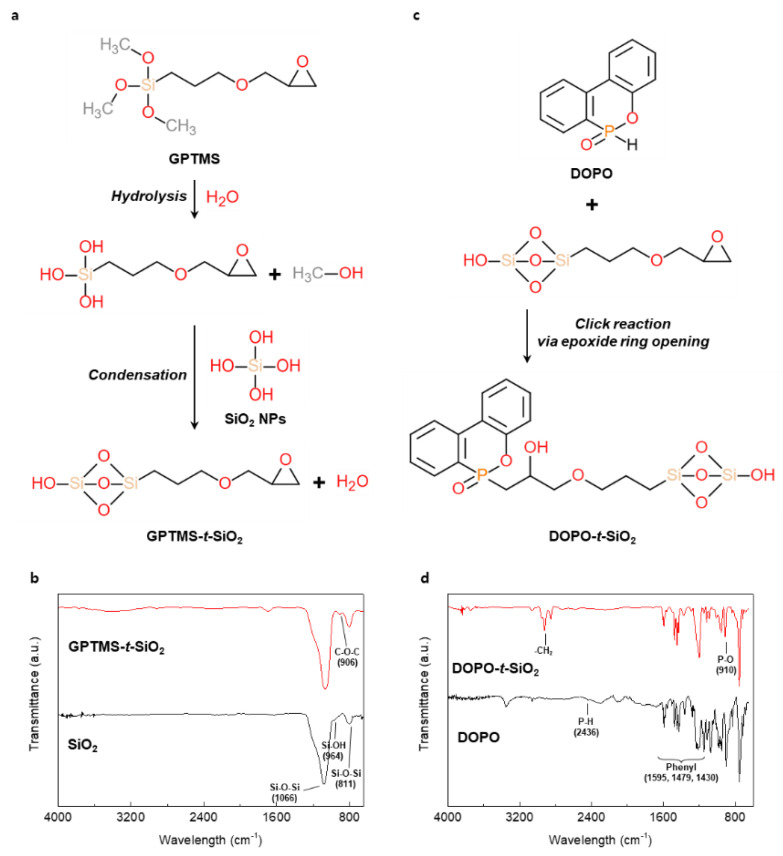
(**a**) Synthesis mechanism and (**b**) Fourier transform infrared (FTIR) spectra of GPTMS-*t*-SiO_2_. (**c**) synthesis mechanism and (**d**) FT-IR spectra of DOPO-*t*-SiO_2_.

**Figure 2:**
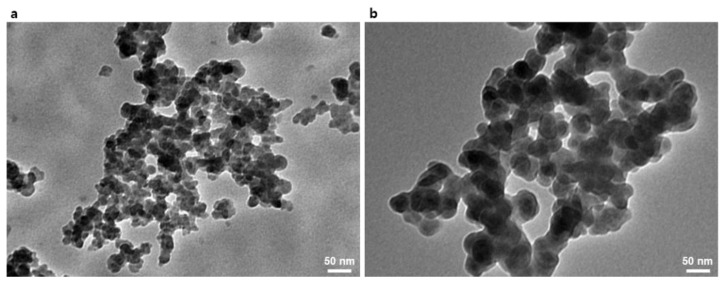
Transmission electron microscopy (TEM) images of (**a**) pristine SiO_2_ nanoparticles (NPs) and (**b**) as-synthesized DOPO-*t*-SiO_2_ NPs.

First, the GPTMS was used to introduce the epoxide moiety to the SiO_2_ NPs, as shown in Figure 1a. The methyl-end groups in GPTMS were transformed to hydroxyl groups by the hydrolysis with the by-product of methanol. The hydrolyzed GPTMS was directly grafted to SiO_2_ NPs through condensation reactions with by-product of water molecules, leading to GPTMS-*t*-SiO_2_ NPs. The interaction during reaction procedure was analyzed using FT-IR, as shown in Figure 1b. For the GPTMS-*t*-SiO_2_ sample, a newly occurred peak at 906 cm^−1^ was assigned to C-O-C from the existence of epoxide group, indicating to successful grafting of GPTMS onto SiO_2_ NPs. Second, the DOPO was attached to enhance flame retardancy of SiO_2_ NPs, as shown in Figure 1c. The synthesized GPTMS-*t*-SiO_2_ NPs were reacted with DOPO by simple click reaction via the opening of epoxide rings in the end of GPTMS, followed by forming P-C bond between DOPO-GPTMS, which was also analyzed using Fourier transform infrared (FTIR), as shown in Figure 1d. For the DOPO-*t*-SiO_2_ sample, the peaks at 2850 and 2900 cm^−1^ were assigned to the symmetric stretching of -CH_2_ group in GPTMS and the peaks at 1595, 1479 and 1430 cm^−1^ were assigned to the existence of phenyl ring from DOPO [42]. Importantly, it was observed that the peak at 2436 cm^−1^ in DOPO assigned to P-H bond was clearly disappeared and the peak at 910 cm^−1^ assigned to P-O bond was maintained after the click reaction, which indicated the successful synthesis of DOPO-*t*-SiO_2_ NPs. It was also observed that the phosphorus peak in EDS spectrum was newly appeared for DOPO-*t*-SiO_2_ NPs after click reaction with fine distribution from aggregates of GPTMS-*t*-SiO_2_ (Figure S1). In addition, the size of pristine SiO_2_ NPs with a diameter of approximately 18 nm was increased by approximately 41 nm after functionalization with DOPO, indicating the DOPO molecules were successfully attached onto the SiO_2_ NPs, as shown in Figure 2.

### 3.2. Mechanical Properties of DOPO-t-SiO_2_ NPs/epoxy Nanocomposites

**Figure 3:**
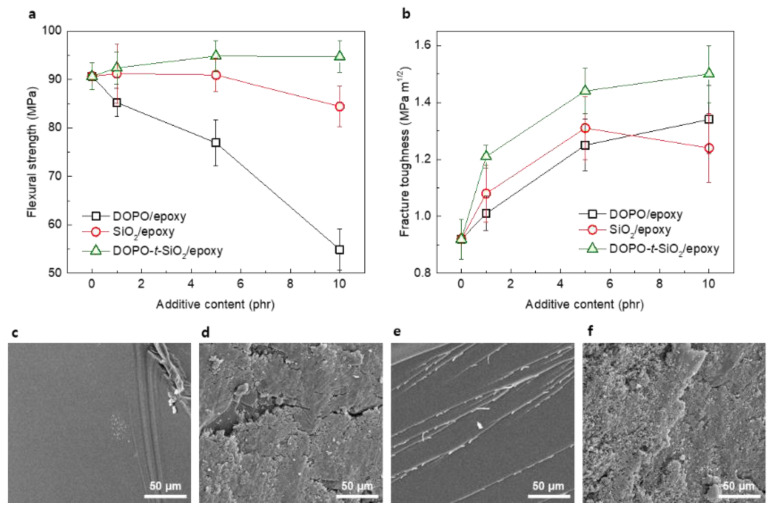
(**a**) Flexural strength and (**b**) fracture toughness of DOPO/epoxy, SiO_2_/epoxy and DOPO-*t*-SiO_2_/epoxy nanocomposites with the content of additives, respectively. SEM images of (**c**) pristine epoxy, (**d**) DOPO/epoxy, (**e**) SiO_2_/epoxy and (**f**) DOPO-*t*-SiO_2_/epoxy nanocomposites, respectively.

We investigated the mechanical properties of epoxy nanocomposites reinforced with as-synthesized DOPO-*t*-SiO_2_ NPs, as shown in Figure 2. Flexural strength of epoxy nanocomposites reinforced with DOPO-*t*-SiO_2_ NPs (DOPO-*t*-SiO_2_/epoxy) was measured, as shown in Figure 3a. Pristine epoxy showed flexural strength value of approximately 90 MPa. By introducing DOPO as an additive, the flexural strength values of the epoxy nanocomposites were rapidly decreased with the content of DOPO due to the softening epoxy matrix. Also, epoxy nanocomposites reinforced with pristine SiO_2_ NPs (SiO_2_/epoxy) exhibited trends of slightly decreasing flexural strength with the content of SiO_2_. Meanwhile, the DOPO-*t*-SiO_2_/epoxy exhibited increased flexural strength values even though the existence of DOPO. It is ascribed that the DOPO-*t*-SiO_2_ efficiently complement degradation of strength of epoxy matrix by covalently combining with SiO_2_ NPs. In addition, the existence of DOPO, which is compatible to epoxy, neighboring SiO_2_ NPs allowed advantageously for the efficient dispersion of SiO_2_ NPs within epoxy matrix, because the functionalization through GPTMS and DOPO contributed to form plenty of -OH groups on the surface of SiO_2_ NPs [36]. It is noteworthy that the DOPO with high polarity further enabled the interaction with epoxy, resulting in increase of flexural strength by reducing the distance between SiO_2_ NPs and polymer chains [43]. We also evaluated the fracture toughness of the composite samples, as shown in Figure 3b. The fracture toughness values of epoxy were significantly improved with an incorporation of each additive, including DOPO, SiO_2_ NPs and DOPO-*t*-SiO_2_ NPs as the increase of their content. Especially, the DOPO-*t*-SiO_2_/epoxy exhibited the highest value on fracture toughness in the whole range of additive content and the value reached approximately 1.5 MPa m^1/2^ with the DOPO-*t*-SiO_2_ of 10 phr. Meanwhile, it is ascribed that the untreated SiO_2_ NPs of higher filler content of 10 phr is hardly dispersed in epoxy matrix, resulting in formation of aggregated SiO_2_ NPs with defects, such as voids [44,45].

The morphological evolutions were studied by observing electron images in cross section after fracture toughness test. As expected, the pristine epoxy exhibited clearly fractured surface, leading to poor impact resistance, as shown in Figure 3c. Meanwhile, the DOPO/epoxy exhibited the fracture surface with rough topology by softening epoxy with DOPO, allowing for dispersing the stress against impact, as shown in Figure 3d. The SiO_2_/epoxy exhibited typical pattern of fracture surface blocking the crack propagation by the SiO_2_ NPs, as shown in Figure 2e. The DOPO-*t*-SiO_2_/epoxy exhibited also rough surface identical to that of DOPO/epoxy and the SiO_2_ NPs linked with DOPO were homogeneously dispersed within the epoxy matrix, as shown in Figure 2f. It is ascribed that the incorporating DOPO-*t*-SiO_2_ NPs possess the synergetic effects of retarding crack propagation as well as plasticizing epoxy matrix.

### 3.3. Flame Retardant Properties of DOPO-t-SiO_2_ NPs/Epoxy Nanocomposites

**Figure 4:**
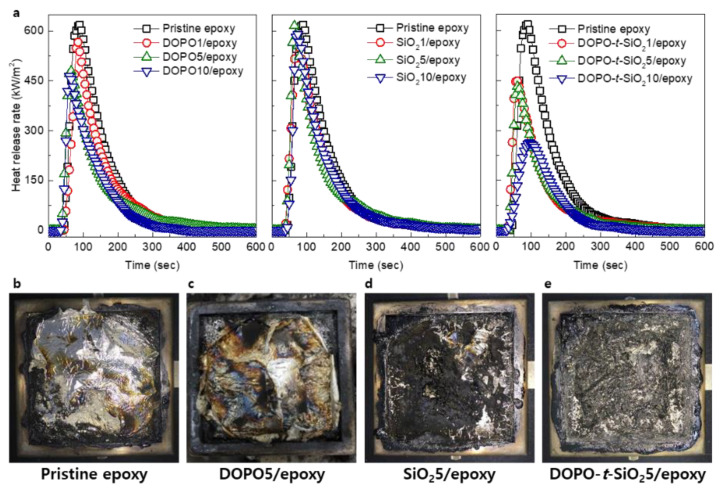
(**a**) Heat release rate measured using cone calorimeter of pristine epoxy, DOPO/epoxy, SiO_2_/epoxy and DOPO-*t*-SiO_2_/epoxy nanocomposites with the content of additives, respectively. Photographs of (**b**) pristine epoxy, (**c**) DOPO5/epoxy, (**d**) SiO_2_5/epoxy and (**e**) DOPO-*t*-SiO_2_5/epoxy nanocomposites after cone calorimeter test, respectively.

The flame-retardant properties of the composite samples were evaluated through cone calorimeter test, in which the heat release rate (HRR), time to ignition (TTI), peak HRR (PHRR), total heat release (THR) and effective heat of combustion (EHC) were obtained, as shown in Figure 4a and Table 1. In the HRR curves as time, the DOPO/epoxy composites exhibited reduced PHRR as well as TTI with the content of DOPO compared to pristine epoxy, which indicate that the DOPO has an important role as flame retardant by effectively reducing their THR. Meanwhile, the SiO_2_/epoxy showed nearly no changes on the HRR curves regardless of the content of SiO_2_ NPs and the PHRR values were dotted with a deviation due to the poor dispersion of untreated SiO_2_ NPs within epoxy. By incorporating the DOPO-*t*-SiO_2_, the epoxy nanocomposite exhibited obviously different behaviors on the HRR, in which the PHRR value was reduced by approximately 60% for the sample with a DOPO-*t*-SiO_2_ of 10 phr that was hardly obtained in the epoxy composites with only a single additive between DOPO or SiO_2_ NPs. It is also noteworthy that SiO_2_ NPs was also advantageous by providing heat resistance effect as well as formation of compact char layer during combustion [46,47]. However, the TTI of DOPO-*t*-SiO_2_/epoxy nanocomposites was slightly fastened as the increased content of SiO_2_ NPs by 5 phr. It is ascribed that the DOPO causes the formation of polyphosphate, giving rise to develop the char from the sample surface by the esterification and dehydrogenation during thermal degradation. The early decomposition of DOPO accelerated the degradation of epoxy matrix at lower temperature and thus ignition energy was significantly reduced [48]. Therefore, the early formation of char was advantageous for flame retardancy of polymer by blocking penetration of oxygen molecules and reducing the ignition energy. Also, the EHC, referring combustion rate of volatile products in the gas phase, was decreased as addition of DOPO with or without SiO_2_ NPs. It is ascribed that the epoxy with DOPO produced non-combustible gas molecules, such as N_2_ and CO_2_, for dilution of flammable gas, while the SiO_2_ NPs hybridized with DOPO facilitated a formation of dense char layer and condensed phase for synergistically improving flame retardancy of the epoxy nanocomposites [49,50]. These behaviors were also coincided from the photograph results of the epoxy and its nanocomposites taken after full ignition. The pristine epoxy showed common appearance by complete burning after ignition with the formation of char at the only edge site, as shown in Figure 4b. As expected, use of DOPO or SiO_2_ NPs additives within epoxy matrix accelerated the formation of char compared to pristine epoxy, as shown in Figure 4c,d. Meanwhile, introducing the DOPO-*t*-SiO_2_ NPs allowed for the uniform generation of char in the whole area, enabled to retardant against burning, as shown in Figure 4e. Consequently, use of DOPO-*t*-SiO_2_ NPs significantly improved the flame retardant properties of the epoxy nanocomposites even without loss of mechanical properties even though the both of properties are in trade-off relationship.

**Figure 5:**
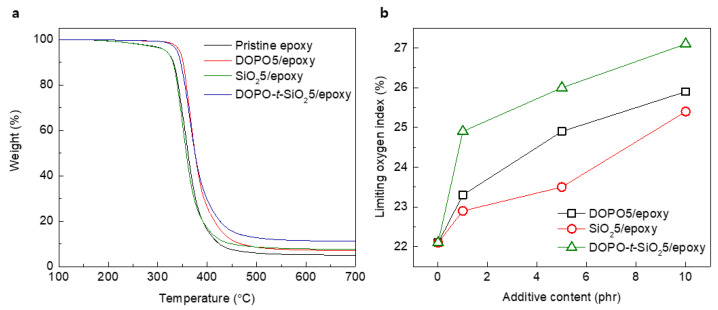
(**a**) Thermogravimetric analysis (TGA) traces of pristine epoxy, DOPO_5_/epoxy, SiO_2_5/epoxy and DOPO-*t*-SiO_2_5/epoxy nanocomposites, respectively. (**b**) Limiting oxygen index plots of DOPO5/epoxy, SiO_2_5/epoxy and DOPO-*t*-SiO_2_5/epoxy nanocomposites, respectively.

From TGA curves, pristine epoxy and SiO_2_5/epoxy nanocomposites exhibited slightly decrease behavior after approximately 200 °C due to the decomposition of epoxy, while the addition of DOPO as flame retardant improved thermal stability by approximately 300 °C, as shown in Figure 5a and Table 2. The char yield which was calculated as the residual weight at 700 °C under a N_2_ atmosphere showed that the well-designed DOPO-*t*-SiO_2_ was effective for formation of char during burning of the epoxy composites rather than the use of other kinds of single-type additives. Also, the LOI values of epoxy composites were totally increased with the content of additives, as shown in Figure 5b. Similar to the above results on flammability test, the DOPO-*t*-SiO_2_5/epoxy nanocomposites exhibited significantly increased LOI values compared to those of the DOPO5/epoxy and SiO_2_5/epoxy nanocomposites. It is ascribed that the flame retardant nature of the DOPO-*t*-SiO_2_5/epoxy nanocomposite arose from the synergetic effects by efficient formation of char restricting the production of combustible gas as well as improved thermal durability through SiO_2_ with lower thermal conductivity [51].

## 4. Conclusions

In this paper, we reported the strategy to design flame retardant hybridized with SiO_2_ and DOPO to allow synergistically flame retardancy for epoxy. DOPO-*t*-SiO_2_ hybrid NPs were successfully synthesized through the surface treatment of SiO_2_ NPs with GPTMS, followed by click reaction with DOPO. The use of DOPO-*t*-SiO_2_ NPs exhibited improved flexural strength of the epoxy nanocomposites, while the values of epoxy nanocomposites reinforced with single additive of DOPO or SiO_2_ NPs were gradually decreased as the content of additive. Also, the fracture toughness of the DOPO-*t*-SiO_2_/epoxy was significantly increased by approximately 1.5 MPa m^1/2^ due to synergetic effects of retardation of crack propagation as well as increased ductility. Moreover, the DOPO-*t*-SiO_2_/epoxy exhibited significantly improved thermal stability and flame retardant properties, in which the PHRR, THR and EHC were obviously reduced, due to not only facile formation of char during ignition but also improved thermal durability by coexistence of covalently combined DOPO and SiO_2_ NPs.

## Figures and Tables

**Table 1:** Data obtained from cone calorimeter test of pristine epoxy, DOPO/epoxy, SiO_2_/epoxy and DOPO-*t*-SiO_2_/epoxy nanocomposites, respectively.

Sample	Additive Content (phr%)	TTI (sec)	PHRR (kW m^−2^)	THR (MJ m^−2^)	EHC (kcal g^−1^)
Pristine epoxy	0	29	618.3	68.9	20.12
DOPO1/epoxy	1	29	566.6	56.2	16.69
DOPO5/epoxy	5	28	479.5	49.7	15.48
DOPO10/epoxy	10	26	463.9	43.8	15.12
SiO_2_1/epoxy	1	28	572.0	58.7	17.53
SiO_2_5/epoxy	5	26	615.9	58.5	16.42
SiO_2_10/epoxy	10	26	590.5	58.6	15.35
DOPO-*t*-SiO_2_1/epoxy	1	23	448.2	43.6	12.23
DOPO-*t*-SiO_2_5/epoxy	5	21	431.5	40.4	11.23
DOPO-*t*-SiO_2_10/epoxy	10	20	269.8	31.4	8.10

**Table 2:** Thermal analysis results of pristine epoxy, DOPO/epoxy, SiO_2_/epoxy and DOPO-*t*-SiO_2_/epoxy nanocomposites, respectively.

Sample	Temperature of 5% Weight Loss (T_d5%_, °C)	Max Decomposition Temperature (°C)	Residual Char at 700 °C (%)
Pristine epoxy	316.3	360.3	5.0
DOPO5/epoxy	344.0	365.3	6.9
SiO_2_5/epoxy	316.6	350.2	7.5
DOPO-*t*-SiO_2_5/epoxy	339.2	364.3	11.1

## Supplementary Materials

The following are available online at https://www.mdpi.com/1996-1944/13/23/5418/s1, Figure S1. SEM and EDS spectrum of (a) GPTMS-*t*-SiO_2_ NPs and (b) DOPO-*t*-SiO_2_ NPs, respectively.
