# Supplementary material for: Thermal and Flame Retardant Properties of Phosphate-Functionalized Silica/Epoxy Nanocomposites
Source: Materials (Basel). 2020 Nov 28;13(23):5418. doi: 10.3390/ma13235418 (PMC7730795; doi:10.3390/ma13235418)
Supplement: Supplementary file 1 [file materials-13-05418-s001.pdf]

materials

Supplementary Materials

Thermal and Flame Retardant Properties of Phosphate-Functionalized Silica/Epoxy Nanocomposites

Il Jin Kim [1,2,†], Jae Wang Ko[1,2,†], Min Seop Song [1], Ji Won Cheon [1,2], Dong Jin Lee [1], Jun Woo Park [3], Seunggun Yu [4,*] and Jin Hong Lee [2,*]

1 New Functional Components Research Team, Korea Institute of Footwear and Leather Technology (KIFLT), Busan 47154, Korea; ijkim@kiflt.re.kr (I.J.K.); jwko@kiflt.re.kr (J.W.K.); sonochemical@gmail.com (M.S.S.); jwcheon@kiflt.re.kr (J.W.C.); almanig84@gmail.com (D.J.L.)

2 Department of Organic Material Science and Engineering, Pusan National University, Busan 46421, Korea; jinhong.lee@pusan.ac.kr

3 Next Generation Battery Research Center, Korea Electrotechnology Research Institute (KERI), Changwon 51543, Korea; jwpark@kiflt.re.kr

4 Insulation Materials Research Center, Korea Electrotechnology Research Institute (KERI), Changwon 51543, Korea; viola@keri.re.kr

* Correspondence: viola@keri.re.kr (S.Y.); jinhong.lee@pnu.ac.kr (J.H.L.)

† These authors are equally contributed.

Received: 29 October 2020; **Accepted**: 25 November 2020; **Published**: 28 November 2020

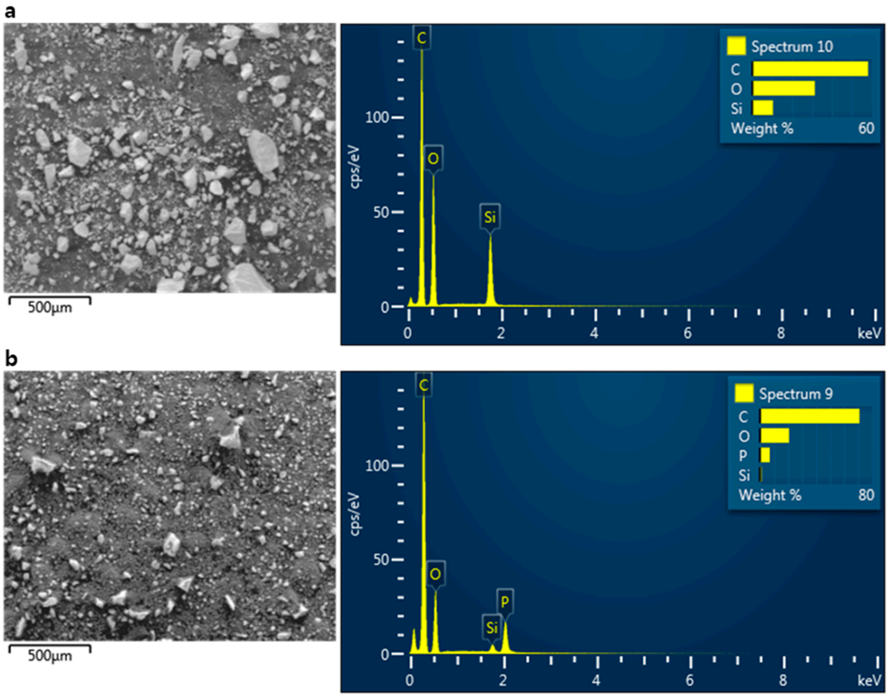

Figure S1. SEM and EDS spectrum of (**a**) GPTMS-*t*-SiO_2 NPs and (**b**) DOPO-*t*-SiO_2 NPs, respectively.
